# A simple model to predict risk of gestational diabetes mellitus from 8 to 20 weeks of gestation in Chinese women

**DOI:** 10.1186/s12884-019-2374-8

**Published:** 2019-07-19

**Authors:** Tao Zheng, Weiping Ye, Xipeng Wang, Xiaoyong Li, Jun Zhang, Julian Little, Lixia Zhou, Lin Zhang

**Affiliations:** 1 Obstetric and Gynecology Department, Xinhua Hospital, Shanghai Jiao Tong University School of Medicine, Shanghai, China; 2 Endocrinology Department, Xinhua Hospital, Shanghai Jiao Tong University School of Medicine, Shanghai, China; 3 MOE-Shanghai Key Lab of Children’s Environmental Health, Xinhua Hospital, Shanghai Jiao Tong University School of Medicine, Shanghai, China; 4 School of Epidemiology and Public Health, Faculty of Medicine, University of Ottawa, Ottawa, Canada

**Keywords:** Gestational diabetes mellitus, Risk prediction, Maternal factors

## Abstract

**Background:**

Gestational diabetes mellitus (GDM) is associated with adverse perinatal outcomes. Screening for GDM and applying adequate interventions may reduce the risk of adverse outcomes. However, the diagnosis of GDM depends largely on tests performed in late second trimester. The aim of the present study was to bulid a simple model to predict GDM in early pregnancy in Chinese women using biochemical markers and machine learning algorithm.

**Methods:**

Data on a total of 4771 pregnant women in early gestation were used to fit the GDM risk-prediction model. Predictive maternal factors were selected through Bayesian adaptive sampling. Selected maternal factors were incorporated into a multivariate Bayesian logistic regression using Markov Chain Monte Carlo simulation. The area under receiver operating characteristic curve (AUC) was used to assess discrimination.

**Results:**

The prevalence of GDM was 12.8%. From 8th to 20th week of gestation fasting plasma glucose (FPG) levels decreased slightly and triglyceride (TG) levels increased slightly. These levels were correlated with those of other lipid metabolites. The risk of GDM could be predicted with maternal age, prepregnancy body mass index (BMI), FPG and TG with a predictive accuracy of 0.64 and an AUC of 0.766 (95% CI 0.731, 0.801).

**Conclusions:**

This GDM prediction model is simple and potentially applicable in Chinese women. Further validation is necessary.

**Electronic supplementary material:**

The online version of this article (10.1186/s12884-019-2374-8) contains supplementary material, which is available to authorized users.

## Background

The prevalence of diabetes in pregnancy, the majority of which is gestational diabetes mellitus (GDM), has been increasing in many jurisdictions [1]. GDM is defined as glucose intolerance that first occurs or is first diagnosed during pregnancy and usually resolves soon after delivery [2]. It raises substantial health concerns both for its short-term adverse effects on pregnancy outcomes and for potential serious long-term consequences for both mothers and their offsprings [2, 3, 4]. For instance, GDM is associated with large for gestational age (LGA), high infant adiposity, shoulder dystocia, caesarean section and preeclampsia [5, 6, 7]. The prevalence of GDM in Chinese women ranged between 13.0 and 20.9%, and the variation was partly due to different criteria [8, 9, 10, 11]. It is important to predict the risk of GDM early in pregnancy to enable early interventions to prevent GDM. Unfortunately, pregnancy is a complex and dynamic process, involving profound changes in energy and nutrient metabolism to sustain fetal development and growth, and to meet the requirements of labour and lactation.

The changes of energy and nutrient metabolism during pregnancy have been postulated to follow a biphasic pattern, predominantly anabolic in early pregnancy and mainly catabolic in later pregnancy [12]. These dynamic changes have not been completely illustrated. For example, in a large cross-sectional study in Israel, triglyceride (TG), total cholesterol (TC) and low density lipoprotein (LDL) levels decreased between the time of conception and 8 weeks of gestation and then gradually increased and peaked just before delivery [13]. However, in a prospective cohort study in Brazil, levels of these lipids increased from the first trimester to the third trimester [14]. Despite the difficulties in risk prediction of GDM due to by these dynamic changes, efforts have been made to develop risk prediction models for GDM incorporating factors such as maternal age, ethnicity, BMI [15], family history of diabetes, personal history of GDM, fasting plasma glucose (FPG), vitamin D3, macrosomia and chronic hypertension, high-sensitive C-reactive protein, placental protein 13, pentraxin 3, myostatin, follistatin, and soluble fms-like tyrosine kinase-1 [15, 16, 17, 18, 19, 20, 21].

It has been reported that the model combining maternal factors including history of GDM, family history of diabetes, ethnicity, parity, BMI, mean arterial pressure (MAP), uterine artery pulsatility index (UtA PI) and pregnancy associated plasma protein A (PAPP-A) in the first trimester predicted GDM with an area under the receiver operating characteristic curve (AUC) of 0.90 (95% CI 0.87–0.92) [22]. Another model developed by applying a machine learning algorithm to data extracted from health records for the first trimester to predict risk GDM at 24–28 weeks of gestation achieved an AUC of 0.86 and accuracy of 62.2%. This algorithm also included maternal factors such as age, parity, BMI, education, FPG and other hematological and biochemical test results [23]. In addition, a model has been developed in obesity pregnant women with age, previous GDM, family history of type 2 diabetes, systolic blood pressure, sum of skinfold thicknesses, waist-to-height ratio, and neck-to-thigh ratio provided an area under the curve of 0.71 for GDM prediction (95% CI 0.68–0.74) [24]. However, it is uncertain whether these models are applicable to Chinese women.

Furthermore, a growing body of evidence suggests that hypertriglyceridaemia is associated with GDM [25, 26]. Hypertriglyceridaemia itself is a well known risk factor for metabolic syndrome. Moreover, it is independently associated with pregnancy outcomes such as LGA, macrosomia [24, 27, 28, 29] and preterm delivery [2, 24]. Whether and to what extent TG level predicts GDM is unknown. Therefore, the purpose of the present study was to develop a simple model incorporating maternal age, BMI, and FPG and TG levels in early pregnancy to predict the risk of GDM.

## Methods

### Participants

**Fig. 1:**
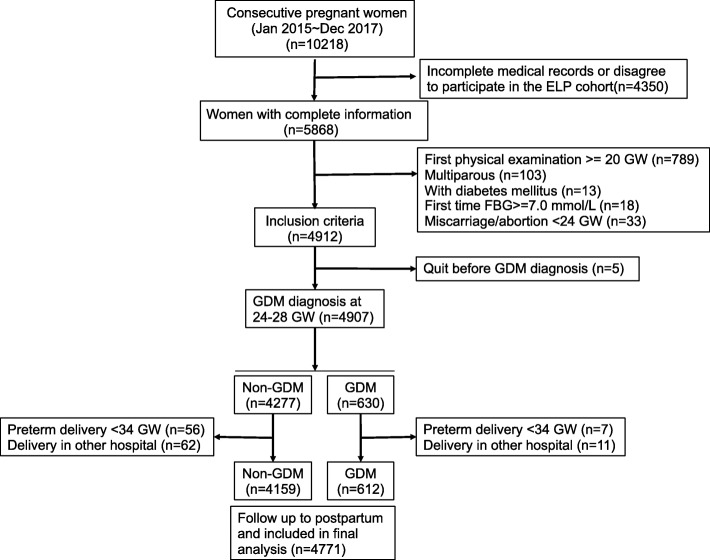
Flowchart of participant recruitment and follow-up

This study was conducted in Xinhua Hospital between Jan 2015 and Dec 2017. Xinhua Hospital is a referral medical center with 3500–4000 deliveries annually. Study participants were recruited in two ways. First, there was retrospective recruitment of consecutive pregnant women delivered from Jan 2015 to Jun 2016, with data obtained solely from medical records. Women were asked before delivery for consent to obtain prenatal clinical records (XHEC-C-2013-001); a total of 2343 women consented and were included in the final analysis. Information abstracted from medical records included pregnancy complications, parity, gestational age at delivery and results of biological indicator, such as FPG, TG, TC, HDL, vitamin D3. Second, women from a prospective hospital-based birth cohort, the Early Life Plan (ELP) study, between Jun 2016 and Dec 2017 (Fig. 1) were also included [30]. Face-to-face informed consent were obtained at the first obstetric visit in the ELP study (XHEC-C-2016-392). For the 2428 women that consented, information on socio-demographic characteristics and medical history, such as diabetes before pregnancy, was collected through a face-to-face interview and self-completed questionnaires. The prenatal clinical information were similar to that obtained from the retrospectively recruited women from medical records. The overall study combined by the two groups of women was approved by the Institutional Review Board of Xinhua hospital (XHEC-C-2018-085).

### Inclusion and exclusion criteria

#### Inclusion criteria

Women were eligible if they: [1] were registered Shanghai residents with no plan to move out of Shanghai in the following two years; [2] had a singleton pregnancy; [3] had their first pregnancy visit prior to 20th week of gestation; [4] planned to give birth in Xinhua hospital; and [5] had complete information on medical history and laboratory results including FPG, glucose challenge test (GCT), fasting plasma lipids, and VitD3 before 20 gestational weeks. The included pregnant women had regularly visits (≥ 6 times) before delivery and followed up until 6 weeks after delivery.

#### Exclusion criteria

Women were excluded if they had any one of the following: [1] multiple pregnancy; [2] type I or type II diabetes mellitus; [3] chronic hypertension; [4] drug treatment of dyslipidaemia before pregnancy; [5] cardiovascular diseases; [6] other diseases that were contraindications of pregnancy.

### Blood measurements

A five milliliter of fasting venous blood sample was taken from pregnant woman on the next day of their first hospital visit. Participants had to be fasting for at least 8 h prior to blood draw. FPG, TC, TG, high density lipoprotein (HDL), LDL, vitamin D_3_ and hemoglobin (Hb) was measured immediately in the hospital laboratory (Hitachi 7180 automatic biochemical analyzer). Coefficients of variance were all less than 5% for the clinic quality assurance.

### Operational definitions

FPG is routinely measured during early pregnancy to detect pre-existing diabetes (FPG ≥ 7 mmol/L). Intermediate level of hyperglycemia is defined as FPG in the range 5.1 to 6.9 mmol/L. [19]

The two-step approach of testing for GDM in this study included an initial screening at 22–24 weeks by administering a 50-g oral glucose solution followed by a 1-h venous glucose test. Women whose glucose levels met or exceeded 7.2 mmol/L underwent a diagnostic 2-h 75-g oral glucose tolerance test (OGTT) between 24 and 26 weeks. The diagnosis of GDM was established when any single glucose concentration met or exceeded a (fasting value of, 5.0 mmol/L or 92 mg/dL; a 1-h value of, 10 mmol/L or 180 mg/dL; or 2-h value, 8.,5 mmol/L or 153 mg/dL) by IADPSG criteria 2010 [31].

Gestational age was determined by ultrasound within 3 months of pregnancy confirmation. Preterm delivery was defined as delivery before 37 weeks of gestation. Sex-specific small for gestational age (SGA) and large for gestational age (LGA) were defined according to the 10th and 90th percentiles of gestational age-specific birth weight in the Chinese population [32]. Recurrent spontaneous abortion was defined as two or more consecutive spontaneous abortions.

### Statistical analyses

Standard descriptive statistics were used to summarize continuous data (mean ± SD as all continuous variables were normally distributed) and categorical data (numbers and percentages). Student’s t test and the chi-square test were used for comparisons between groups. A partial correlation analysis was performed for maternal factors (using the “ppcor” package implemented in R) (https://cran.r-project.org/web/packages/ppcor/ppcor.pdf). Odds ratio of individual maternal factor as a potential predictor for GDM was estimated through logistic regression. Continuous potential predictors were categorized as the “U-type” response is common in biological systems. Such predictors were included in the model as categorical variables when the association between continuous variables and GDM are not linear. As a sensitivity analysis, we examined AUC for the model including these predictors as continuous variables.

Because our analysis included multiple biomarkers that might be correlated, and correlated variables in multivariate regression model may cause problems in estimation of coefficients, we investigated the correlation between FPG, Hb and TG using partial correlation analysis. As we found significant correlations among all participants (further details in section 3.2), we decided to use multivariable logistic regression through Bayesian inference. Thus, the predictive maternal factors were further selected through Bayesian adaptive sampling by multivariate logistic regression (using the “BAS” package implemented in R) (https://cran.r-project.org/web/packages/BAS/BAS.pdf). The selected potentially predictive maternal factors were incorporated into the multivariate Bayesian logistic regression using Markov Chain Monte Carlo simulation (iteration = 20,000, burn-in = 3000) and the Metropolis-Hastings algorithm (using the “MCMC” package implemented in R) (https://cran.r-project.org/web/packages/mcmc/mcmc.pdf). An AUC curve was calculated to estimate the predictive probability and to compare the performance of models. The AUC and optimal cut-off value of the predicted probability of GDM were analyzed (using the “pROC” package) (https://cran.r-project.org/web/packages/pROC/pROC.pdf). To assess the effect of substituting continuous variables for categorical variables in the sensitivity analysis, we compared the difference in AUC using Delong’s ROC curve test [33]. All the analyses were performed with R version 3.3.3 (www.R-project.org). For frequentist analyses, the *p* < 0.05 threshold was regarded as statistically significant unless indicated otherwise.

## Results

### Baseline maternal characteristics and perinatal outcomes

A total 5868 pregnant women provided written informed consent (Fig. 1), of whom 89.6% were in their first (*n* = 605) or second (*n* = 4653) trimester of pregnancy. All participants were Asian. The prevalence of hyperglycemia was 12.7% (743/5868). The number of women who met the inclusion criteria and remained in the study up to the time of GDM screening was 4907; the incidence of GDM diagnosed between 24 and 28 weeks of gestation was 12.8% (630/4907). Among 4907 women, 63 women delivered earlier than 34 weeks and 73 delivered in other hospitals, resulting in 4771 in the final analysis. Just over half (52.8%) of the women were nulliparous and around 0.4% had a history of recurrent spontaneous abortion. Compared with women who did not have GDM, women with GDM were older (mean age 1.8 years higher), had higher prepregnancy BMI and level of FPG, TG, TC and lower HDL values (Table 1). There were no significant differences in LDL, polipoprotein A (ApoA), apolipoprotein B (ApoB), or lipoprotein A (LipoA) between the two groups.

**Table 1:** Baseline maternal characteristics (< 20 GW) and perinatal outcomes among patients with or without GDM

	Total (*n* = 4771) mean (SD) or n(%)	Non-GDM (*n* = 4159) mean (SD) or n(%)	GDM (*n* = 612) mean (SD) or n(%)	*p* value*
Age, (year)	30.4 (4.0)	30.2 (3.9)	32.0 (4.2)	< 0.0001
Prepregnancy BMI, (kg/m^2^)	22.5 (4.3)	22.3 (4.2)	24.1 (4.7)	< 0.0001
Nullipara	2521 (52.8)	2239 (54.0)	282 (46.1)	< 0.0001
Repeated spontaneous abortion	22 (0.5)	16 (0.4)	6 (1.0)	0.0870
FPG, (mM)	4.69 (0.41)	4.64 (0.37)	5.02 (0.51)	< 0.0001
TG, (mM)	1.45 (0.67)	1.41 (0.64)	1.68 (0.80)	< 0.0001
TC, (mM)	4.72 (0.80)	4.71 (0.79)	4.79 (0.82)	0.0310
HDLc, (mM)	2.02 (0.71)	2.04 (0.73)	1.87 (0.51)	< 0.0001
LDLc, (mM)	2.26 (0.59)	2.25 (0.59)	2.31 (0.56)	0.1058
AopA, (g/l)	1.82 (0.31)	1.83 (0.30)	1.77 (0.35)	0.0936
ApoB, (g/l)	0.79 (1.27)	0.77 (1.19)	1.00 (1.78)	0.1890
LipoA, (mg/dl)	16.3 (17.2)	16.4 (17.5)	14.9 (13.7)	0.2959
Gestational week at inclusion	14.2 (1.74)	14.2 (1.74)	14.0 (1.75)	0.0028
Preterm delivery (34 < GW < 37)	197 (4.1)	166 (4.0)	31 (5.1)	0.2551
Gestational week at delivery	39.1 (1.16)	39.1 (1.17)	38.8 (1.01)	< 0.0001
Birth weight, (g)	3328.2 (428.5)	3330.3 (422.8)	3313.9 (465.8)	0.4103
Birth weight > 4000 (g)	267 (5.6)	231 (5.5)	36 (5.9)	0.8223
SGA	263 (5.5)	231 (5.5)	32 (5.2)	0.8164
AGA	4073 (85.4)	3564 (85.7)	509 (83.2)	0.1122
LGA	421 (8.8)	352 (8.5)	69 (11.3)	0.0269
Birth height, (cm)	49.4 (2.43)	49.5 (2.15)	49.1 (3.88)	0.0444
Hospital stay, (day)	6.3 (5.2)	6.1 (5.1)	8.4 (6.1)	< 0.0001
Vaginal delivery Forceps	2353 (49.3) 36 (0.8)	2132 (51.3) 28 (0.7)	221 (36.1) 8 (1.3)	< 0.0001 0.0090
Caesarean Section	2382 (49.9)	1999 (48.1)	383 (62.6)	< 0.0001
Neonatal jaundice	1294 (27.1)	1184 (28.5)	110 (18.0)	0.0024
Apgar score at 1 min	9.94 (0.70)	9.95 (0.74)	9.94 (0.35)	0.7386
NICU admission	258 (5.4)	220 (5.3)	38 (6.2)	0.3921
Neonatal death	4 (0.1)	3 (0.1)	1 (0.1)	0.4219

Note: Baseline maternal plasma glucose, lipids and lipoproteins were all determined before 20 weeks of gestation

*, *p* value of Student t test for continuous data or chi-square test for categorical data in comparison between non-GDM and GDM groups

Women with GDM had a slightly but significant earlier delivery than non-GDM women, but the proportion of preterm delivery between 34 and 37 weeks did not differ between these two groups (even when premature delivery before 34 weeks were included, *p* = 0.422). Compared women without GDM, women with GDM had higher proportion of LGA, length of hospital stay and proportion of deliveries by caesarean section were increased in GDM women, but had lower neonatal height and lower prevalence of neonatal jaundice (Table 1). The proportions of birth weight greater than 4000 g, neonatal intensive care unit (NICU) admission and neonatal death were not different between these two groups.

### Metabolic maternal factors are correlated

**Fig. 2:**
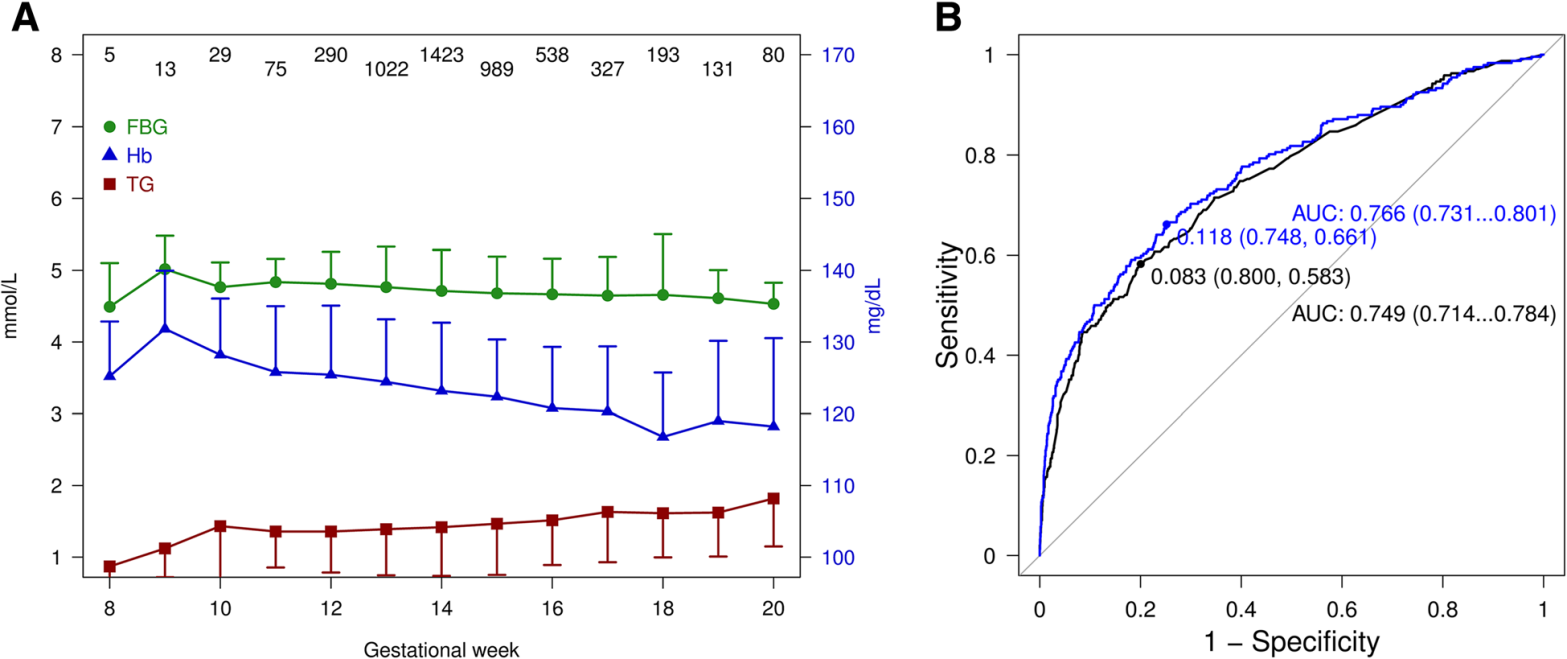
a Levels of FPG and TG by gestational age between 8th and 20th week Number of participants at each gestational week indicated at the top of the figure. Figure 2b ROC curve of GDM risk prediction models based on multivariate logistic regression. The black curve was derived from regression model using categorical variables as indicated in Table 3 (rightmost column). The curve in blue was derived from model following Bayesian variable selection and model averaging and, this best fitted model using maternal age, pre-gravid BMI, FBG, TG, TC and LDL as predictive variables. The predicted optimal cut-off probabilities to discriminate GDM from non-GDM mothers for categorical variable model and Bayesian variable selected model were 0.083 (with specificity = 0.80 and sensitivity = 0.58) and 0.118 (with specificity = 0.75 and sensitivity = 0.66), respectively. The AUR was presented as averaged level and 95% CI in the same color

FPG levels slightly decreased with gestational age between 8th and 20th weeks, while TG levels slightly increased (Fig. 2A). The slopes of linear regression between FPG and TG with gestational age were − 0.0245 (*p* < 0.0001) and 0.0444 (*p* < 0.0001), respectively. Among all participants, after adjusting for maternal age, prepregnancy BMI and gestational age at first visit, FPG was correlated with Hb, vitamin D3 and lipids except apolipoprotein B and lipoprotein A. The correlation patterns differed between women with or without GDM. Among those with GDM, FPG was only correlated with Hb and HDL (Table 2).

**Table 2:** Partial correlation between maternal fasting plasma glucose (FPG) e, hemoglobulin, triglycerides and vitamin D3

	FPG at first visit					
	Total (n = 4771)		Non-GDM (n = 4159)		GDM (n = 612)	
	R’	*p* value	R’	*p* value	R’	*p* value
Hb	0.1075	< 0.0001	0.0829	< 0.0001	0.1849	< 0.0001
TG	−0.0358	0.0247	−0.1003	< 0.0001	0.0833	0.0582
TC	0.0314	0.0479	0.0271	0.1124	0.0266	0.5446
HDL	−0.1454	< 0.0001	− 0.1327	< 0.0001	−0.1987	0.0020
LDL	−0.1351	< 0.0001	−0.1667	< 0.0001	− 0.0277	0.6689
ApoA	−0.0724	0.0271	−0.0280	0.0580	−0.1874	0.0580
ApoB	0.0193	0.5562	0.0052	0.8820	0.0428	0.6676
LipoA	−0.0142	0.6676	−0.0041	0.9083	−0.0560	0.5846
VitD3	0.1476	< 0.0001	0.1580	< 0.0001	0.0244	0.6364

Note: The Pearson partial correlation between fasting plasma glucose, hemoglobulin and lipids were adjusted by maternal age, prepregnancy BMI and gestational age at the first visit

Hb, hemoglobulin; TG, triglyceride, TC, total cholesterol, HDL, high densitylipoprotein; LDL, low density lipoprotein; ApoA, apolipoprotein A; ApoB, apolipoprotein B, LipoA, lipoprotein A; VitD3, vitamin D3

### Predicting GDM risk between 8th to 20th gestational weeks (including validation ROC)

Factors that were statistically significantly associated with GDM in univariate logistic regression were included in the multivariable logistic regression. After we applied Bayesian adaptive sampling, maternal age, prepregnancy BMI, FPG and TG were selected because they showed high density interval (HDI) of adjusted odds ratio (OR) that did not include 1.0 (Table 3). In this multivariable logistic regression analysis, women who aged between 18 and 29 years, had prepregnancy BMI less than 27, FPG less than 4.4 mM and TG less than 2.26 mM were less likely to develop GDM. The risk of GDM increased with maternal age, FPG and TG.

**Table 3:** Independent and adjusted ORs of potential factors following logistic regression analysis of GDM

	Univariate logistic regression (n = 4771)		Multivariate logistic regression using Bayesian inference (*n* = 4771)
	OR (95% CI)	*p* value	ORa (95% HDI)
Age, 18~30 years	reference		
30~35	1.83 (1.48, 2.27)	< 0.0001	1.38 (1.01, 1.88)
35–40	2.53 (1.94, 3.30)	< 0.0001	1.69 (1.06, 2.63)
40–45	3.72 (2.18, 6.36)	< 0.0001	3.03 (1.06, 7.82)
Prepregnancy BMI, 16~23 kg/m^2^	reference		
23–27.5	1.92 (1.56, 2.36)	< 0.0001	1.17 (0.83, 1.59)
27.5–30	5.17 (3.53, 7.56)	< 0.0001	3.39 (1.92, 5.68)
30~35	4.26 (2.63, 6.91)	3.39 (1.92, 5.68)	1.96 (0.98, 4.09)
35~45	2.64 (1.41, 4.96)	0.0025	0.32 (0.02, 2.70)
FPG, 2.0~4.4 mM	reference		
4.4~5.1	2.56 (1.72, 3.81)	< 0.0001	2.44 (1.54, 4.33)
5.1~7.0	21.16 (13.89, 32.23)	< 0.0001	16.58 (9.21, 31.30)
TG 0~1.69 mM	reference		
1.69~2.26	1.75 (1.38, 2.21)	16.58 (9.21, 31.30)16.58 (9.21, 31.30)	1.40 (0.92, 1.92)
2.26~12.0	3.04 (2.31, 4.00)	< 0.0001	2.10 (1.45, 3.56)
Abnormal gravidity outcome (0 time)	reference		
≥1 time#	1.49 (1.25, 1.77)	< 0.0001	1.05 (0.75, 1.41)
TC, 0~5.2 mM	reference		
5.2~10	1.23 (1.01,1.50)	0.0381	0.88 (0.58, 1.31)
HDL, ≥1.0 mM	reference		
< 1.0	3.34 (1.17, 9.57)	0.0246	2.67 (0.83, 8.56)
LDL, 0~2.6 mM	reference		
2.6~12	1.15 (0.19, 6.89)	0.361	1.15 (0.77, 1.69)

Note: ORa, adjusted OR; only the lower cut off value was included in each subgroup. CI, confidence interval; HDI, high density interval; #, at least one preterm delivery miscarriage or induced abortion;

The AUC of the final GDM predictive model that incorporated categorical maternal age, prepregnancy BMI, FPG and TG was 0.749 (95% CI 0.714, 0.784). In the sensitivity analysis including these risk factors as continuous rather than categorical variables in the model, the AUC was 0.766 (95% CI 0.731, 0.801). These two models were not significantly different (Delong’s ROC curve test *p* = 0.49) (Fig. 2B).

Next, we estimated sensitivity, specificity, positive predictive value (PPV), negative predictive value (NPV) of the final model. When median specificity was held at 0.80 the sensitivity was at 0.60~0.61 and the cut-off value of probability was 0.160~0.161 using metric variables of maternal age, prepregnancy BMI, FPG and TG in model. The sensitivity of the model with categorical variables was 0.59 and cut-off probability was 0.130~0.131 (Fig. 2B). We then validated the cut-off probabilities using the original dataset, 0.160 for metric and 0.130 for categorical variable models. The consistencies of diagnosis could be 0.64 and 0.63 for metric and categorized risk models while the PPVs were 0.32 and 0.31 and both NPVs were 0.93. The following formula was developed to predict the probability of GDM between 8th and 20th gestational weeks, based on maternal age, prepregnancy BMI, FPG, and TG:

Probability of GDM = 1/(1 + 1/exp. (Age*0.0601 + BMI*0.03405 + FPG*2.46427 + TG*0.30648–16.87528)).

A reference probability table of various sensitivities and specificities is also provided for future validation (Additional file 2: Table S1).

## Discussion

The association of unusual rise of FPG and TG with GDM and/or pregnancy outcomes had been explored [5, 10, 11, 13, 15, 26]. FPG ≥5.1 mmol/L predicted LGA risk [34] and anti-diabetic treatment (e.g., insulin, metformin) might reduce the risk of adverse perinatal outcomes [35]. Zhao et al. went even further to try to quantitatively explore these associations by logistic regression [36]. If the predictors are correlated as in this case, it is improper to use multivariable logistic regression by frequentist’s method. Liu et al. might have realized this pitfall of using logistic regression, but still they failed to show that FPG correlated with TG or other lipids in their analysis [28]. There are other GDM predictive tools incorporating more biochemical or ultrasonic maternal factors. To our knowledge, the model developed in this study using information on maternal factors such as age, prepregnancy BMI, FPG and TG and using robust modeling methods is the first model applicable to Chinese women for whom no ethnicity-specific GDM risk prediction model has been available. Further validation of the model is necessary before generalizing it to the vast rural population of China.

The incidence of GDM in this study (12.8%) was lower than the overall frequency (17.8%) reported in HAPO study [37]. The prevalence of GDM was much lower in studies that used either the WHO 1980–2013 or ADA 2002–2014 criteria (13.0 to 13.9%) than in studies that used IADPSG and China MOH criteria which gave a prevalence of 19.9 and 20.9%, respectively [8]. The prevalence of GDM also differed by screening methods, with the one-step screening method yielding a prevalence of GDM of 14.7%, and the two-step screening method a prevalence of 7.2%, about half that of the one-step method [8, 38, 39].The prevalence of GDM would have been higher if the lower fasting and post-load glucose thresholds for GDM diagnosis which was previously applied in south Asian women (from 17.4 to 24.2%) had been used [5]. In a systematic review, the GDM was postulated to affect 14% of pregnant women annually and accounted for 90% of hyperglycemia pregnancies [40].

Women with GDM also had elevated FPG and TG levels in the early stage of pregnancy, and as a consequence had an increased risk of adverse perinatal outcomes such as high birth weight (e.g., macrosomia or LGA) and caesarean section [28, 33]. In a consensus guideline the Australasian Diabetes in Pregnancy Society (ADIPS) has urged more research to identify the most appropriate tests for detecting GDM in early pregnancy. Only following early detection can risk stratification and early intervention for GDM become possible, but still it is difficult. The available models suffer from one or more of the limitations including applying only to sub-populations [24], involving complicated algorithms [23], requiring specific laboratory tests [21, 22, 24] or applying in specific periods before GDM diagnosis [21, 22, 41]. The model derived in the present study did not have AUC, sensitivity or specificity as high as some previous models but it was simple, applicable to the Chinese population and covered a broad period before GDM diagnostic tests, and therefore has the potential to be widely used in less developed jurisdictions.

Risk factors for GDM included a history of hyperglycemia, having a first degree relative with diabetes mellitus, a history of macrosomia, polycystic ovarian syndrome, age > 40 years, pre-pregnancy BMI > 30 kg/m [2], and taking medications such as corticosteroids, and antipsychotics [15, 17, 40, 41]. This list is increasing. Physically active Asian women have a reduced risk of GDM (OR = 0.56, 95% CI 0.32–0.98), especially those who were overweight (OR = 0.52, 95% CI 0.29–0.93) or obese (OR = 0.34, 95% CI 0.15–0.77) [42]. Weight gain has been reported to be an independent risk factor of GDM [43], especially in the first three months of pregnancy. Levels of C-reactive protein (hs-CRP) and sex hormone binding globulin (SHBG) in the first trimester may predict GDM with high sensitivity [44]. Blood count had been incorporated in a GDM prediction model [45]. Hemoglobin A1C (HbA1c) level may be helpful in GDM diagnosis, especially in the third trimester [46, 47]. Correlations between glucose and lipid levels, together with interactions among potential risk factors, further complicate GDM prediction. How to develop a reliable GDM risk prediction model by using the above-mentioned complicated risk factors poses a challenge. Regression analysis using Bayesian inference is applicable to predictors that are independent or correlative. However, principal component analysis and other methods are also approaches to combine correlated or interacting predictors. We used Bayesian inference in regression analysis because it has a similar interpretative framework to frequentist logistic regression but is more robust when correlated predictors are present. In the sensitivity analysis using frequentist logistic regression, the ORs and AUC, sensitivity and specificity were very similar to those obtained by Bayesian inference (Additional file 3: Table S2, Additional file 2: Table 2, Additional file 1: Figure S1).

We also classified the continuous variables such as maternal age, pre-pregnancy BMI, FPG, TG, TC, HDL, LDL etc. into categories according to clinical reference intervals for healthy adults. In terms of predicted risk probability and AUC, the model based on categorical variables did not show higher predictive performance than the model based on continuous variables (AUC 0.748 vs 0.770, *p* = 0.316). The categorical variables did not show significant interaction effects. Fasting glucose and triglycerides may increase with gestation weeks. The original full model also included a history of abnormal gravidity (preterm birth, miscarriage and induced abortion). Following variable selection the best model incorporated four factors including maternal age, prepregnancy BMI, FPG and TG. The pseudo-R-squared was 0.165 for the best fit model, suggesting that the potential risk factors included could accounted only for a small fraction of the variance of GDM. Further studies on GDM risk factors and prediction models should pay more attention to understanding the interactions among the risk factors.

The strengths of this study include a moderate sample size, a robust modeling strategy for correlated predictors, and its development of a simple formula for prediction based on only four maternal factors (age, prepregnancy BMI, FPG and TG). This formula can be applied even if only a calculator is available, such as in less-developed parts of China. The limitations are that we were not able to incorporate all known potential risk factors in modeling, that the participants came from a single medical center, and that we have not yet conducted external validation. Due to intrinsic nature of observational data in this cohort study, the internal validity was low to moderate.

## Conclusions

From 8th to 20th gestational week, the average FPG level indicated a slight decreasing trend while, in line with previous studies, TG levels displayed a slight increasing trend. The metabolites were intercorrelated during this period of pregnancy. We developed a prediction model in Chinese women, which addressed the correlation of predictors and incorporated maternal age, prepregnancy BMI, FPG and TG, with a predictive accuracy of 0.64 and an AUC of 0.766 (95% CI 0.731, 0.801). Thus, a simple model, which can be applied using a hand calculator, may predict the risk of GDM and be used in less economically developed parts of China.

## Additional files

Additional file 1: **Figure S1.** Trace and density of estimated coefficients of final multivariate logistic models obtained using Markov Chain Monte Carlo simulation. The Markov Chain Monte Carlo logistic regression was conducted using the Metropolis iteration (*n* = 20,000) with Burn-in = 3000 (with default value of MCMClogit in MCMCpack library). Left column panel displays the variation in parameters of the included variables while the right column panel displays summaries of the distribution of the parameters (PDF 585 kb)

Additional file 2: **Table S1.** Thresholds of predicted GDM probability and corresponding specificity and sensitivity. Probability of GDM = 1/(1 + 1/exp. (Age*0.0601 + BMI*0.03405 + FPG*2.46427 + TG*0.30648–16.87528)) (PDF 1779 kb)

Additional file 3: **Table S2.** Odds ratios of GDM associated with risk factors selected for inclusion in model, cutoff probability at maximal AUC, AUC, sensitivity and specificity estimated using frequentist method and Bayesian inference (PDF 1436 kb)

## Abbreviation

ADA: The American Diabetes Association
ADIPS: The Australasian Diabetes in Pregnancy Society
ApoA: Apolipoprotein A
ApoB: Apolipoprotein B
AUC: Area under receiver operating characteristic curve
BAS: Bayesian adaptive sampling
BMI: Body mass index
CI: Confidence interval
CRP: C-reactive protein
ELP: Early Life Plan
FPG: Fasting plasma glucose
GCT: Glucose challenge test
GDM: Gestational diabetes mellitus
HAPO: Hyperglycemia And Adverse Pregnancy Outcome
Hb: Hemoglobin
HbA1c: Glycated hemoglobin
HDI: High density interval
HDL: High density lipoprotein
IADPSG: The International Association of the Diabetes and Pregnancy Study Groups
LDL: Low density lipoprotein
LGA: Large for gestational age
LipoA: Lipoprotein A
MAP: Mean arterial pressure
MCMC: Markov Chain Monte Carlo simulation
MOH: Ministry Of Health
NICU: Neonatal intensive care unit
NPV: Negative predictive value
OGTT: Oral glucose tolerance test
OR: Odds ratio
PAPP-A: Pregnancy associated plasma protein A
PPV: Positive predictive value
pROC: Partial Receiver operating characteristic
ROC: Receiver operating characteristic
SD: Standard deviation
SGA: Small for gestational age
SHBG: Sex hormone binding globulin
TC: Total cholesterol
TG: Triglyceride
UtA PI: Uterine artery pulsatility index

## References

[1] American Diabetes Association (2018). Management of diabetes in pregnancy: standards of medical Care in Diabetes-2018. Diabetes Care.

[2] Reece EA, Leguizamon G, Wiznitzer A (2009). Gestational diabetes: the need for a common ground. Lancet (London, England).

[3] Catalano PM, McIntyre HD, Cruickshank JK, McCance DR, Dyer AR, Metzger BE (2012). The hyperglycemia and adverse pregnancy outcome study: associations of GDM and obesity with pregnancy outcomes. Diabetes Care.

[4] Farrar D, Simmonds M, Bryant M, Sheldon TA, Tuffnell D, Golder S (2016). Hyperglycaemia and risk of adverse perinatal outcomes: systematic review and meta-analysis. Bmj.

[5] Farrar D, Fairley L, Santorelli G, Tuffnell D, Sheldon TA, Wright J (2015). Association between hyperglycaemia and adverse perinatal outcomes in south Asian and white British women: analysis of data from the born in Bradford cohort. Lancet Diabetes Endocrinol.

[6] World Health Organization (2014). Diagnostic criteria and classification of hyperglycaemia first detected in pregnancy: a World Health Organization guideline. Diabetes Res Clin Pract.

[7] Albrecht SS, Kuklina EV, Bansil P, Jamieson DJ, Whiteman MK, Kourtis AP (2010). Diabetes trends among delivery hospitalizations in the U.S., 1994-2004. Diabetes Care.

[8] Lee KW, Ching SM, Ramachandran V, Yee A, Hoo FK, Chia YC (2018). Prevalence and risk factors of gestational diabetes mellitus in Asia: a systematic review and meta-analysis. BMC Pregnancy and Childbirth.

[9] Chu SY, Abe K, Hall LR, Kim SY, Njoroge T, Qin C (2009). Gestational diabetes mellitus: all Asians are not alike. Prev Med (Baltim).

[10] Jin W-Y, Lin S-L, Hou R-L, Chen X-Y, Han T, Jin Y (2016). Associations between maternal lipid profile and pregnancy complications and perinatal outcomes: a population-based study from China. BMC Pregnancy Childbirth.

[11] Shen H, Liu X, Chen Y, He B, Cheng W (2016). Associations of lipid levels during gestation with hypertensive disorders of pregnancy and gestational diabetes mellitus: a prospective longitudinal cohort study. BMJ Open.

[12] Herrera E (2002). Lipid metabolism in pregnancy and its consequences in the fetus and newborn. Endocrine.

[13] Wiznitzer A, Mayer A, Novack V, Sheiner E, Gilutz H, Malhotra A (2009). Association of lipid levels during gestation with preeclampsia and gestational diabetes mellitus: a population-based study. Am J Obstet Gynecol.

[14] Farias DR, Franco-Sena AB, Vilela A, Lepsch J, Mendes RH, Kac G (2016). Lipid changes throughout pregnancy according to pre-pregnancy BMI: results from a prospective cohort. BJOG.

[15] Wang C, Zhu W, Wei Y, Su R, Feng H, Lin L (2016). The predictive effects of early pregnancy lipid profiles and fasting glucose on the risk of gestational diabetes mellitus stratified by body mass index. J Diabetes Res.

[16] Zhang Y, Gong Y, Xue H, Xiong J, Cheng G (2018). Vitamin D and gestational diabetes mellitus: a systematic review based on data free of Hawthorne effect. BJOG: An International Journal of Obstetrics & Gynaecology.

[17] Geraghty AA, Alberdi G, O’Sullivan EJ, O’Brien EC, Crosbie B, Twomey PJ (2017). Maternal and fetal blood lipid concentrations during pregnancy differ by maternal body mass index: findings from the ROLO study. BMC Pregnancy Childbirth.

[18] Spracklen CN, Smith CJ, Saftlas AF, Robinson JG, Ryckman KK (2014). Maternal hyperlipidemia and the risk of preeclampsia: a meta-analysis. Am J Epidemiol.

[19] Cosson E, Carbillon L, Valensi P (2017). High fasting plasma glucose during early pregnancy: a review about early gestational diabetes mellitus. J Diabetes Res.

[20] Smirnakis Karen V., Plati Alicia, Wolf Myles, Thadhani Ravi, Ecker Jeffrey L. (2007). Predicting gestational diabetes: choosing the optimal early serum marker. American Journal of Obstetrics and Gynecology.

[21] Zhao B, Han X, Meng Q, Luo Q (2018). Early second trimester maternal serum markers in the prediction of gestational diabetes mellitus. J Diabetes Investig.

[22] Sweeting AN, Wong J, Appelblom H, Ross GP, Kouru H, Williams PF (2018). A first trimester prediction model for gestational diabetes utilizing aneuploidy and pre-eclampsia screening markers. J Matern Fetal Neonatal Med.

[23] Qiu H, Yu H-Y, Wang L-Y, Yao Q, Wu S-N, Yin C (2017). Electronic health record driven prediction for gestational diabetes mellitus in early pregnancy. Sci Rep.

[24] White SL, Lawlor DA, Briley AL, Godfrey KM, Nelson SM, Oteng-Ntim E (2016). Early antenatal prediction of gestational diabetes in obese women: development of prediction tools for targeted intervention. PLoS One.

[25] Ryckman KK, Spracklen CN, Smith CJ, Robinson JG, Saftlas AF (2015). Maternal lipid levels during pregnancy and gestational diabetes: a systematic review and meta-analysis. BJOG.

[26] Ghodke B, Pusukuru R, Mehta V (2017). Association of Lipid Profile in pregnancy with preeclampsia, gestational diabetes mellitus, and preterm delivery. Cureus.

[27] Kulkarni SR, Kumaran K, Rao SR, Chougule SD, Deokar TM, Bhalerao AJ (2013). Maternal lipids are as important as glucose for fetal growth: findings from the Pune maternal nutrition study. Diabetes Care.

[28] Liu B, Geng H, Yang J, Zhang Y, Deng L, Chen W (2016). Early pregnancy fasting plasma glucose and lipid concentrations in pregnancy and association to offspring size: a retrospective cohort study. BMC Pregnancy Childbirth.

[29] Boghossian NS, Mendola P, Liu A, Robledo C, Yeung EH (2017). Maternal serum markers of lipid metabolism in relation to neonatal anthropometry. J Perinatol.

[30] Zhang J, Tian Y, Wang W, Huang H, Shen X, Sun K (2016). Toward a national birth cohort study in China. Am J Public Health.

[31] Metzger BE, Gabbe SG, Persson B, Buchanan TA, Catalano PA, Damm P (2010). International association of diabetes and pregnancy study groups recommendations on the diagnosis and classification of hyperglycemia in pregnancy. Diabetes Care.

[32] Zhu L, Zhang R, Zhang S, Shi W, Yan W, Wang X (2015). [Chinese neonatal birth weight curve for different gestational age]. *Zhonghua er ke za zhi = Chinese*. J Pediatr.

[33] DeLong ER, DeLong DM, Clarke-Pearson DL (1988). Comparing the areas under two or more correlated receiver operating characteristic curves: a nonparametric approach. Biometrics..

[34] Noussitou P, Monbaron D, Vial Y, Gaillard RC, Ruiz J (2005). Gestational diabetes mellitus and the risk of metabolic syndrome: a population-based study in Lausanne, Switzerland. Diabetes Metab.

[35] Farrar D, Simmonds M, Bryant M, Sheldon TA, Tuffnell D, Golder S (2017). Treatments for gestational diabetes: a systematic review and meta-analysis. BMJ Open.

[36] Zhao M, Li GH. The value of fasting plasma glucose and lipid profiles between 7 and 15 gestational weeks in the prediction of gestational diabetes mellitus. Zhonghua Fu Chan Ke Za Zhi. 2016;25;51(11):835–9. [Article in Chinese]. 10.3760/cma.j.issn.0529-567X.2016.11.007 27916067

[37] Sacks DA, Hadden DR, Maresh M, Deerochanawong C, Dyer AR, Metzger BE (2012). Frequency of gestational diabetes mellitus at collaborating centers based on IADPSG consensus panel-recommended criteria: the hyperglycemia and adverse pregnancy outcome (HAPO) study. Diabetes Care.

[38] Song L, Shen L, Li H, Liu B, Zheng X, Zhang L, Xu S, Wang Y (2017). Socioeconomic status and risk of gestational diabetes mellitus among Chinese women. Diabet Med.

[39] Leng J, Liu G, Zhang C, Xin S, Chen F, Li B (2016). Physical activity, sedentary behaviors and risk of gestational diabetes mellitus: a population-based cross-sectional study in Tianjin, China. Eur J Endocrinol.

[40] Tieu J, Mcphee AJ, Crowther CA, Middleton P. Screening and subsequent management for gestational diabetes for improving maternal and infant health. Cochrane Database Syst Rev. 2014;(2014):CD007222. 10.1002/14651858.CD007222.pub3 24515533

[41] Melchior H, Kurch-Bek D, Mund M (2017). The prevalence of gestational diabetes. Dtsch Arztebl Int.

[42] Padmapriya N, Bernard JY, Liang S, Loy SL, Cai S, Zhe IS (2017). Associations of physical activity and sedentary behavior during pregnancy with gestational diabetes mellitus among Asian women in Singapore. BMC Pregnancy Childbirth.

[43] Ludwig DS, Currie J (2010). The association between pregnancy weight gain and birthweight: a within-family comparison. Lancet (London, England).

[44] Maged AM, Moety GAF, Mostafa WA, Hamed DA (2014). Comparative study between different biomarkers for early prediction of gestational diabetes mellitus. J Matern Fetal Neonatal Med.

[45] Sahbaz A, Cicekler H, Aynioglu O, Isik H, Ozmen U (2016). Comparison of the predictive value of plateletcrit with various other blood parameters in gestational diabetes development. J Obstet Gynaecol.

[46] Renz PB, Cavagnolli G, Weinert LS, Silveiro SP, Camargo JL (2015). HbA1c test as a tool in the diagnosis of gestational diabetes mellitus. PLoS One.

[47] Ho Y-R, Wang P, Lu M-C, Tseng S-T, Yang C-P, Yan Y-H (2017). Associations of mid-pregnancy HbA1c with gestational diabetes and risk of adverse pregnancy outcomes in high-risk Taiwanese women. PLoS One.

