# Supplementary material for: A simple model to predict risk of gestational diabetes mellitus from 8 to 20 weeks of gestation in Chinese women
Source: BMC Pregnancy Childbirth. 2019 Jul 19;19:252. doi: 10.1186/s12884-019-2374-8 (PMC6642502; doi:10.1186/s12884-019-2374-8)
Supplement: Supplementary file 1 — Figure S1. Trace and density of estimated coefficients of final multivariate logistic models obtained using Markov Chain Monte Carlo simulation. The Markov Chain Monte Carlo logistic regression was conducted using the Metropolis iteration (n = 20,000) with Burn-in = 3000 (with default value of MCMClogit in MCMCpack library). Left column panel displays the variation in parameters of the included variables while the right column panel displays summaries of the distribution of the parameters (PDF 585 kb) [file 12884_2019_2374_MOESM1_ESM.pdf]

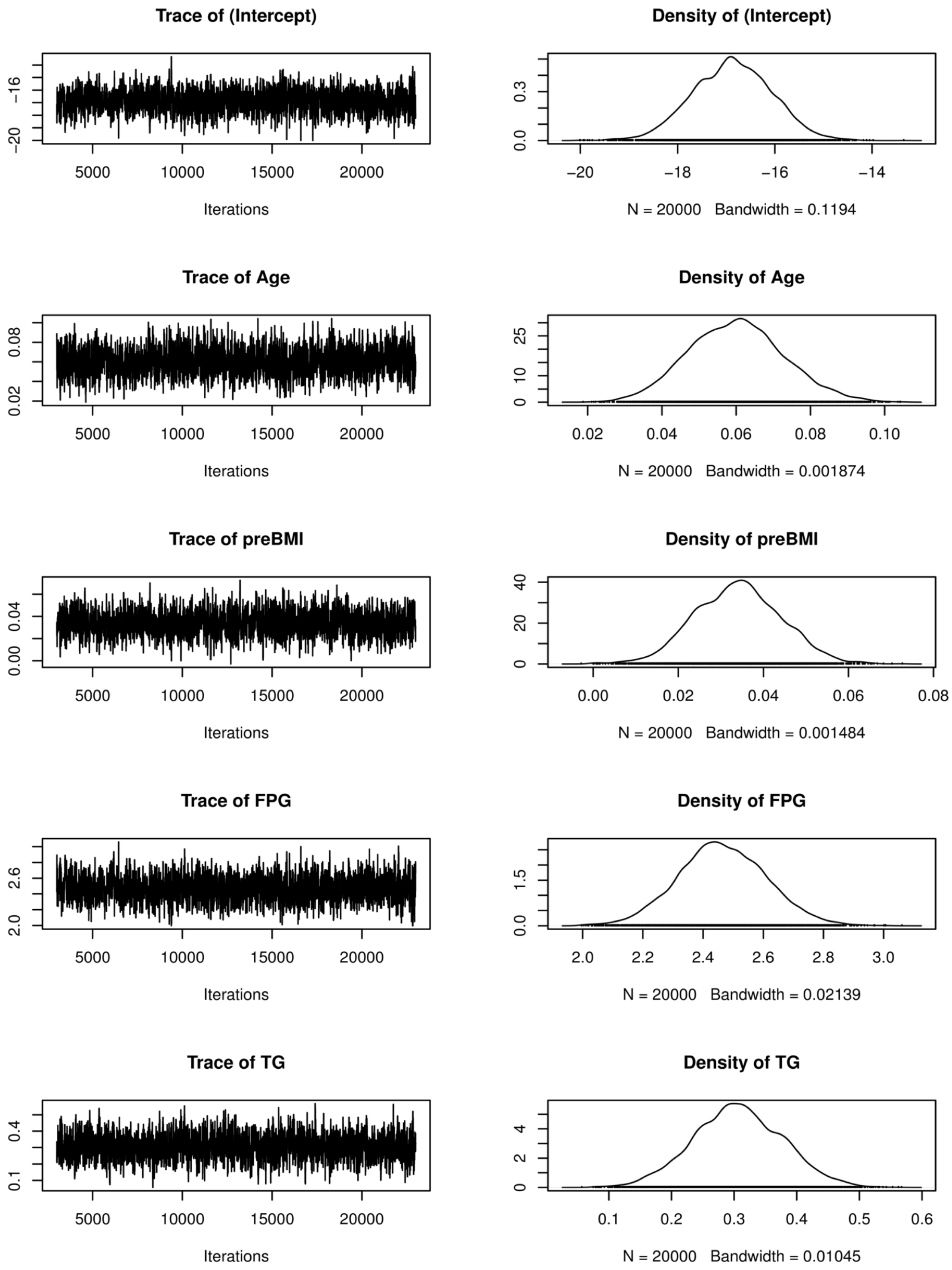
Trace of (Intercept)
Density of (Intercept)
Iterations
N = 20000 Bandwidth = 0.1194
Trace of Age
Density of Age
Iterations
N = 20000 Bandwidth = 0.001874
Trace of preBMI
Density of preBMI
Iterations
N = 20000 Bandwidth = 0.001484
Trace of FPG
Density of FPG
Iterations
N = 20000 Bandwidth = 0.02139
Trace of TG
Density of TG
Iterations
N = 20000 Bandwidth = 0.01045
