# Supplementary material for: A simple model to predict risk of gestational diabetes mellitus from 8 to 20 weeks of gestation in Chinese women
Source: BMC Pregnancy Childbirth. 2019 Jul 19;19:252. doi: 10.1186/s12884-019-2374-8 (PMC6642502; doi:10.1186/s12884-019-2374-8)
Supplement: Supplementary file 2 — Table S1. Thresholds of predicted GDM probability and corresponding specificity and sensitivity. Probability of GDM = 1/(1 + 1/exp. (Age*0.0601 + BMI*0.03405 + FPG*2.46427 + TG*0.30648–16.87528)) (PDF 1779 kb) [file 12884_2019_2374_MOESM2_ESM.pdf]

Cut-off Probability	Specificity (low)	Specificity (median)	Specificity (high)	Sensitivity (low)	Sensitivity (median)	Sensitivity (high)
0.0379	0.1603	0.1734	0.1857	0.9309	0.9501	0.9674
0.0552	0.2969	0.3115	0.3273	0.8752	0.9002	0.9251
0.0747	0.4292	0.4455	0.4613	0.8196	0.8503	0.8810
0.0823	0.4876	0.5048	0.5203	0.7965	0.8292	0.8618
0.0893	0.5328	0.5504	0.5664	0.7658	0.8004	0.8349
0.1004	0.5927	0.6093	0.6260	0.7121	0.7505	0.7889
0.1105	0.6339	0.6499	0.6663	0.6910	0.7294	0.7678
0.1224	0.6794	0.6955	0.7107	0.6621	0.7006	0.7409
0.1397	0.7358	0.7512	0.7655	0.6257	0.6641	0.7044
0.1438	0.7472	0.7618	0.7758	0.6104	0.6507	0.6929
0.1597	0.7883	0.8018	0.8146	0.5739	0.6161	0.6564
0.1674	0.8029	0.8158	0.8286	0.5566	0.6008	0.6411
0.1827	0.8394	0.8511	0.8631	0.5355	0.5758	0.6161
0.1937	0.8569	0.8683	0.8794	0.5086	0.5509	0.5912
0.2167	0.8876	0.8972	0.9074	0.4587	0.5010	0.5432
0.2422	0.9139	0.9226	0.9314	0.4107	0.4530	0.4952
0.2690	0.9299	0.9381	0.9460	0.3570	0.4012	0.4415
0.2868	0.9428	0.9501	0.9574	0.3301	0.3724	0.4127

Note: not for clinical diagnosis, only for research.

Probability of GDM=1/(1+1/exp(Age*0.0601+BMI*0.03405+FPG*2.46427+TG*0.30648-16.87528)).
