# Supplementary material for: A simple model to predict risk of gestational diabetes mellitus from 8 to 20 weeks of gestation in Chinese women
Source: BMC Pregnancy Childbirth. 2019 Jul 19;19:252. doi: 10.1186/s12884-019-2374-8 (PMC6642502; doi:10.1186/s12884-019-2374-8)
Supplement: Supplementary file 3 — Table S2. Odds ratios of GDM associated with risk factors selected for inclusion in model, cutoff probability at maximal AUC, AUC, sensitivity and specificity estimated using frequentist method and Bayesian inference (PDF 1436 kb) [file 12884_2019_2374_MOESM3_ESM.pdf]

	Frequentist		Bayesian
	**OR (95% CI)**	***p* value**	**OR (94% HDI)**
Age	1.06(1.03,1.09)	2.219e-6	1.06(1.04,1.09)
Pre-gravid BMI	1.03(1.01, 1.05)	7.454e-4	1.03(1.01,1.05)
FPG	11.74(8.79,15.68)	1.759e-62	11.75(8.85,15.74)
TG	1.36(1.18, 1.56)	1.190e-5	1.36(1.18,1.56)
Cutoff probability at maximal AUROC	0.178	NA	0.178
AUROC	0.766 (0.73, 0.80)	NA	0.766 (0.73, 0.80)
Sensitivity	0.587	NA	0.589
Specificity	0.842	NA	0.843

Note: FPG, fasting plasma glucose; TG, triglyceride; cutoff prob., cutoff probability using the model formula and original dataset; AUROC, area under the receiver operating curve.
